# Warfare-related Complex Abdominal Wall Reconstruction Using a Bioprosthetic Regenerate Template and Negative Pressure Therapy

**Published:** 2009-05-16

**Authors:** Jacob J. Glaser, Forest R. Sheppard, Fred A. Gage, Anand R. Kumar, William A. Liston, Eric A. Elster, James R. Dunne, Charles L. Blankenship

**Affiliations:** ^a^ Department of Surgery, National Naval Medical Center, Bethesda, MD 20889; ^b^ Uniformed Services, University of the Health Sciences, Bethesda, MD 20814; ^c^ Regenerative Medicine Department, Naval Medical Research Center, Silver Spring, MD 20910; ^d^ Department of Plastic and Reconstructive Surgery, National Naval Medical Center, Bethesda, MD 20889.

## Abstract

The views expressed in this article are those of the authors and do not reflect the official policy of the Department of the Army, Department of the Navy, the Department of Defense, or the United States Government.

We are military service members (or employees of the US Government). This work was prepared as part of our official duties. Title 17 U.S.C. 105 provides that “Copyright protection under this title is not available for any work of the United States Government.” Title 17 U.S.C. 101 defines a US Government work as a work prepared by a military service member or employee of the US Government as part of that person's official duties.

Warfare-related torso/abdominal wounds are often unique and complex and can pose a significant reconstructive challenge. The objective of this manuscript is to report the unique and successful management of a complex warfare-related abdominal wound. A dermal regenerate template in combination with negative pressure wound therapy was used to reconstitute lateral abdominal wall integrity after radical debridement and control of a necrotizing soft tissue infection of the torso. Adjunctive continuous negative pressure (vacuum assisted closure) therapy was used to provide external coverage and encourage the formation of granulation tissue. With this combination therapy, torso wound size decreased in surface area by 82% and the underlying musculofascial defect decreased by 64%. Neovascularization of a 55-cm^2^ acellular dermal graft was achieved as evidenced by surface granulation and complete survival of a partial-thickness skin graft. In our patient with a complex war injury, advanced tissue replacement techniques and negative pressure wound therapy resulted in a decreased abdominal wall defect, a restoration of abdominal wall integrity/domain, and allowed for concurrent surgical treatment of complex intra-abdominal injuries.

Current military operations result in unique high-energy tissue trauma due to improvised explosive devices, assault rifle projectiles, and motor vehicle accidents. These massive soft-tissue and alimentary tract injuries carry a high potential for morbidity and mortality and require multidisciplinary care.^1^ The concurrent use of a dermal regenerate template and negative pressure therapy for the successful management of abdominal wall defects with concurrent necrotizing soft tissue infection and pancreatic trauma, requiring debridement and external drainage, has not previously been described. The optimal management of these difficult wounds remains poorly characterized. In this report, we describe unique and successful management of a complex, warfare-related abdominal wound.

## CASE REPORT

**Figure 1:**
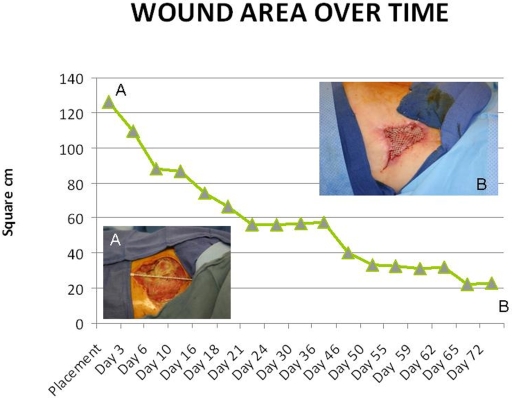
Point A represents initial evaluation of wound with wound area measurements. Plotted points represent area of wound measured intraoperatively. Point B measures area of wound at day of skin graft placement.

A 36-year-old active duty marine sustained massive torso trauma by sniper fire while on routine patrol. The patient's Injury Severity Score was 50 at presentation. Prior to casualty evacuation (CASEVAC) to the continental United States, the patient underwent a left anteriolateral thoractomy, a nonanatomic resection of the left lower lobe of the lung, exploratory celiotomy with subsequent diaphragm repair, splenectomy, subtotal colectomy, creation of an end ileostomy, repair of gastrotomies, and a cholecystectomy. During evacuation, a necrotizing soft tissue infection of his right flank at the bullet exit site developed, which required radical full-thickness debridement of the right lateral abdominal wall. The patient arrived to the National Naval Medical Center 96 hours after initial injury in critical condition requiring full ventilatory support, with an open midline celiotomy incision, and a lateral abdominal wall defect in need of acute management (Fig 1).

**Figure 2:**
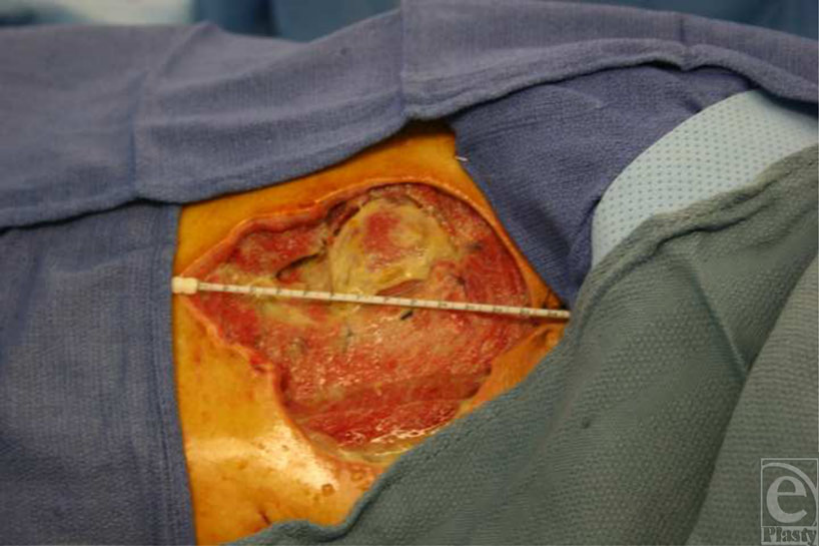
A 55-cm^2^ full-thickness defect of the lateral abdominal musculature and fascia upon initial presentation.

After stabilization in the surgical intensive care unit, the patient underwent operative abdominal exploration. The patient was noted to have a previously unrecognized pancreas injury with peripancreatic inflammation, necrosis, and saponification of the body and tail of the gland requiring partial necrosectomy and external drainage. A 14.9 cm × 13.3 cm wound of the right flank with a 55-cm^2^ full-thickness defect of the lateral abdominal musculature and fascia existed (Fig 2). The defect was approximately 4 cm lateral and cephalad relative to the end ileostomy. The presence of the flank wound made control of intra-abdominal drainage difficult and created a significant risk for the development of an enterocutaneous fistula. The large lateral abdominal wall defect was reconstructed with an onlay cellular dermal regenerate template (eg, AlloDerm, LifeCell, Branchburg, NJ) secured to the remnants of the lateral abdominal wall musculature with a running 2-0 PDS suture. Immediate coverage of the implant was obtained using a negative pressure dressing (eg, wound V.A.C. Therapy, KCI, San Antonio, Tex) set to 125 mm~Hg of continuous negative pressure. With this reconstruction, the exposed bowel was effectively covered re-creating the lateral abdominal wall. The injured pancreas was then controlled with standard wide drainage and the ileostomy was diverted from the wound with a stoma appliance. The central celiotomy defect was then sequentially closed.

**Figure 3:**
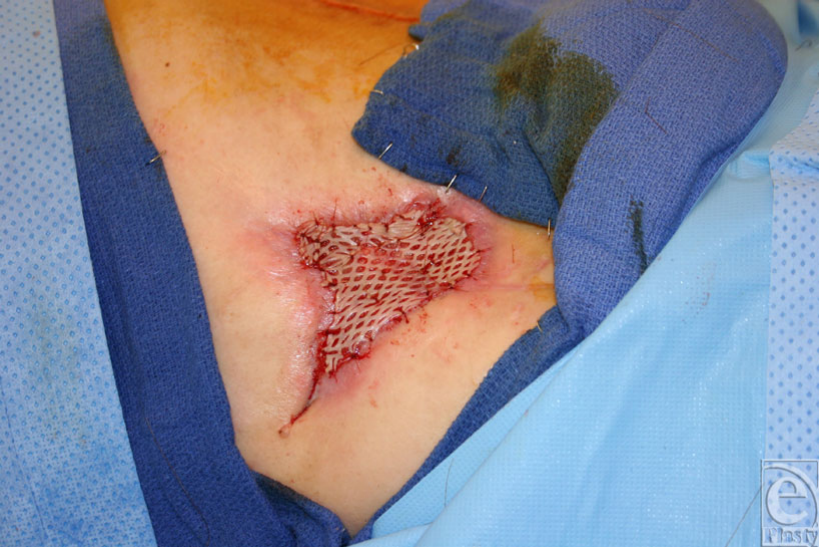
A partial-thickness skin graft was used for coverage of vascularized AlloDerm bed.

**Figure 4:**
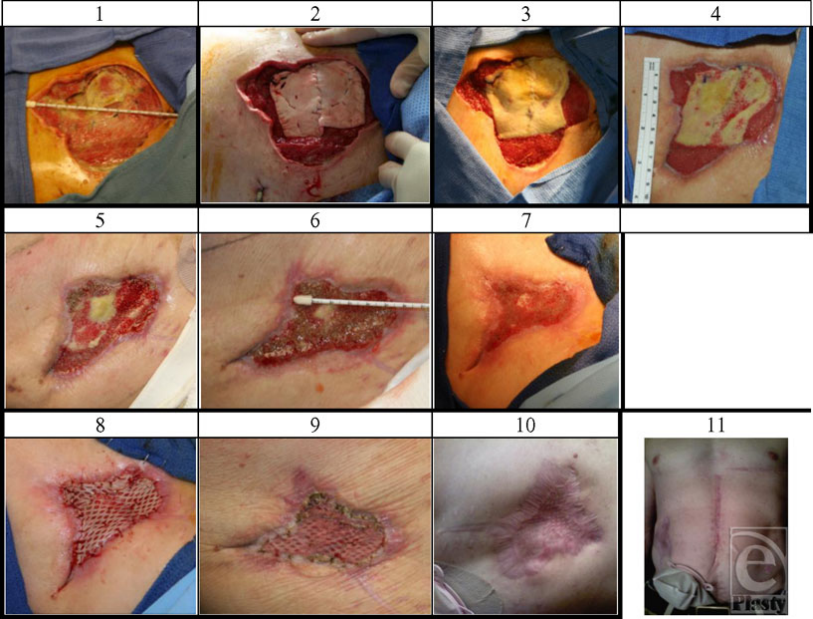
(1) Initial wound; (2) after washout out and AlloDerm applied to wound; (3) day 10 after AlloDerm placement; (4) day 33 after AlloDerm placement; (5) day 49 after AlloDerm placement; (6) day 69 after AlloDerm placement; (7) day 72 after AlloDerm placement; (8) day 72 after skin graft; (9) 5 days after skin graft; (10) 208 days after skin graft; and (11) 290 days postinjury.

The lateral abdominal wall reconstruction using a combination of AlloDerm and negative pressure dressing (V.A.C.) was managed with serial dressing changes in the operating room at 96-hour intervals. The size of the wound decreased significantly, and after 2 weeks the V.A.C. changes were done at the bedside at 5-day intervals. He was able to transition to outpatient treatment with a portable wound V.A.C. and undergo rehabilitation at his home duty station. After 72 days of continuous therapy with constant negative pressure, the wound had decreased in size by 82%, the underlying fascial defect decreased by 64%. The exposed AlloDerm demonstrated neovascularization manifest by complete coverage with granulation tissue. A partial-thickness skin graft was used for coverage of the remaining open wound with complete take of the graft on the vascularized AlloDerm bed (Figs 3 and 4). No cutaneous, subcutaneous, or sub-AlloDerm infectious complications occurred during the course of treatment, and empiric antibiotic coverage for this wound, or its reconstruction, was not utilized.

At the time of this report, the patient is 9 months status postreconstruction. Abdominal contour is good with no overt “bowing out” of AlloDerm with moderate valsalva; however, a palpable 20-cm^2^ abdominal wall defect, by clinical examination, is present underlying otherwise healthy skin (Fig 4). Currently the patient is awaiting restoration of intestinal continuity and will subsequently undergo definitive reconstruction of the remaining defect.

## DISCUSSION

High-energy warfare-related abdominal wounds present many unique challenges to military surgeons. Stabilization of the patient is performed near the battlefield followed by transfer to sequentially higher levels of care as permitted by operational considerations. Definitive surgical management of complex wounds does not begin until several days after the initial injury.^1^ Thus, warfare-related injured patients present to the continental US Military Treatment Facility 3–5 days after initial injury often with open and still-contaminated wounds that require intensive management.

Negative pressure wound therapy (V.A.C.) has become an essential component in the treatment of these patients at our institution. V.A.C. has proven to be effective for treatment of chronic wounds and of soft tissue injuries associated with trauma.^2^ Removing edema, encouraging revascularization of the wound bed, and stimulating cellular proliferation V.A.C. have become a useful adjunct in wound therapy.^3^ V.A.C. has been used successfully in contaminated warfare-related wounds as well.^4^ In addition, the V.A.C. dressing in conjunction with skin grafts has significantly improved graft survival in problematic wounds.^5^

Human Acellular Dermis (AlloDerm) has been used successfully for the treatment of problematic ventral hernias.^6^ AlloDerm has been used to reconstruct large abdominal wall defects and is advantageous over prosthetic mesh primarily in the setting of heavy contamination.^7^ For complex abdominal wall defects, AlloDerm can be used to achieve a tension-free repair with the potential for revascularization.^8^

Uniquely coupling the properties of the wound V.A.C. and AlloDerm, we have demonstrated durable abdominal wall reconstruction while treating concurrent visceral injuries. After stabilization of the patient's visceral injuries, his nutrition status improved and the lateral abdominal wall defect began contracting. AlloDerm was revascularized enough to support a partial-thickness skin graft within 66 days. The portable nature of the wound V.A.C. allowed the patient to undergo physical therapy and rehabilitation while on continuous negative pressure therapy and to have his wounds managed in an outpatient setting. At the time of wound closure with partial-thickness skin grafting, his wound and fascial defect demonstrated significant contraction.

Complicated abdominal wall defects associated with visceral injury in severely injured patient remains challenging. An optimal strategy for wound closure and management, which facilitates treatment of other life-threatening injuries, has not been well described. The use of a dermal regenerate template coupled with negative pressure wound therapy in this case facilitated visceral injury management and reconstructed the abdominal wall defect in a critically ill patient. Though intended as an initial damage control maneuver, it resulted in the closure of a 126-cm^2^ abdominal wound and revascularization of a 55-cm^2^ AlloDerm graft and facilitated closure of the wound within 66 days of placement. This technique proved essential to this patient's recovery and may be applicable to more clinical scenarios than previously thought.
